# GCEN: An Easy-to-Use Toolkit for Gene Co-Expression Network Analysis and lncRNAs Annotation

**DOI:** 10.3390/cimb44040100

**Published:** 2022-03-25

**Authors:** Wen Chen, Jing Li, Shulan Huang, Xiaodeng Li, Xuan Zhang, Xiang Hu, Shuanglin Xiang, Changning Liu

**Affiliations:** 1 State Key Laboratory of Developmental Biology of Freshwater Fish, School of Life Sciences, Hunan Normal University, Changsha 410081, China; chenwen@biochen.org (W.C.); huangshulan5920@163.com (S.H.); deng18397646718@163.com (X.L.); huxiang@hunnu.edu.cn (X.H.); 2 CAS Key Laboratory of Tropical Plant Resources and Sustainable Use, Xishuangbanna Tropical Botanical Garden, Chinese Academy of Sciences, Kunming 650223, China; lijing3@xtbg.ac.cn (J.L.); zhangxuan@xtbg.ac.cn (X.Z.); 3 Core Botanical Gardens, Center of Economic Botany, Chinese Academy of Sciences, Menglun 666303, China; 4 The Innovative Academy of Seed Design, Chinese Academy of Sciences, Kunming 650223, China

**Keywords:** gene co-expression network, lncRNAs annotation, RNA-Seq, gene ontology, KEGG

## Abstract

Gene co-expression network analysis has been widely used in gene function annotation, especially for long noncoding RNAs (lncRNAs). However, there is a lack of effective cross-platform analysis tools. For biologists to easily build a gene co-expression network and to predict gene function, we developed GCEN, a cross-platform command-line toolkit developed with C++. It is an efficient and easy-to-use solution that will allow everyone to perform gene co-expression network analysis without the requirement of sophisticated programming skills, especially in cases of RNA-Seq research and lncRNAs function annotation. Because of its modular design, GCEN can be easily integrated into other pipelines.

## 1. Introduction

Long non-coding RNAs (lncRNAs) are generally defined as transcripts with more than 200 nucleotides but without protein-coding potential. A variety of lncRNAs play import roles in diverse biological processes, such as human diseases [1], innate immunity [2], abnormal development [3], and so on. The recent advances in RNA-Seq technology have immensely boosted the discovery of abundant lncRNAs across many species. The characterization of the functions of lncRNAs are accruing but still lag behind expectations. Unlike protein-coding genes, lncRNAs are often less conserved at the level of primary sequence [4]. As a result, traditional gene annotation approaches, which are based on the similarity of sequence or structure and have achieved great success in protein-coding genes, have become impracticable in the exploration of lncRNAs function.

In contrast, the guilt-by-association approaches, which use associations or interactions between genes to extract functional meaning rather than sequence similarity analysis, provide a feasible strategy for lncRNAs function annotation [5]. The gene co-expression network is one such method which is generally constructed according to high-throughput gene expression profiles from microarray or RNA-Seq. The assumption of gene annotation by a co-expression network is that genes with similar expression pattern are likely to share similar functions [6]. Existing studies have revealed that lncRNAs often execute functions with their protein partners, and coding-lncRNA gene co-expression network analysis has been widely used to identify the potential functions of novel lncRNAs in human [7] and in many model organisms [5,8].

At present, there are some tools or web servers that provide gene co-expression network analysis and/or lncRNAs function annotation services. For example, WGCNA is an R-based tool for gene co-expression network construction and module identification mainly for microarray data [9]. The server ncFANs is a co-expression-network based webservice for lncRNAs function annotation [10]. However, there are certain new situations to consider for RNA-Seq and coding-lncRNA gene co-expression network analysis, such as data normalization and network construction methods [11], as well as the computational challenges of much larger datasets when lncRNA genes are considered. In addition, it is valuable to run such calculations efficiently on a local computer in many scenarios. Thus, we require an effective cross-platform toolkit for providing all services with network construction and module identification, which has been proven to be effective in many applications on function prediction and annotation of lncRNAs [5,8].

In this work, we present GCEN, a cross-platform command-line toolkit developed with C++, which can be used by biologists to easily build a gene co-expression network and predict gene function. The toolkit GCEN is an efficient and easy-to-use solution that can be easily integrated into other pipelines due to its modular design. It will allow everyone to perform gene co-expression network analysis without the requirement of sophisticated programming skills, especially in RNA-Seq research or in lncRNAs function annotation.

## 2. Materials and Methods

The program GCEN was developed using C++ as an open-source software under the GPLv3 license. In addition to our code, we used some third-party code in compliance with their licenses. The program GCEN can be compiled and run in Linux, Windows, and macOS. Compiling GCEN requires a compiler and library support for the ISO C++ 2011 standard. No new computational methods or algorithms are used in GCEN, rather we implemented and integrated some classic algorithms to annotate lncRNA faster and more easily. We describe the main algorithms used as follows.

### 2.1. Data Normalization

We implemented five algorithms, namely, median normalization, quantile normalization [12], the median-of-ratios method [13], trimmed mean of M-values (TMM) [14], and housekeeping genes normalization. For the median normalization, the median of gene expression values in each sample is calculated, and then each gene expression value in the same sample is multiplied by the mean of all medians and divided by the median of this sample. For housekeeping genes normalization, we adjusted the gene expression values so that the median of the housekeeping genes expression in each sample was the same. The descriptions of the other three algorithms can be found in their original papers.

### 2.2. Co-Expression Network Construction

We used the Spearman or Pearson correlation coefficients directly to determine co-expression patterns between gene pairs, which are better performing and more robust [11]. The coefficient of Spearman or Pearson correlation  ρ was calculated as follows.

(1) $$\rho \;=\;\frac{\sum_{i}\left(x_{i}-\bar{x}\right)\left(y_{i}-\bar{y}\right)}{\sqrt{\sum_{i}{\left(x_{i}-\bar{x}\right)}^{2}\sum_{i}{\left(y_{i}-\bar{y}\right)}^{2}}}$$

For Pearson correlation, x or y represents the vector of the expression value of each gene, $x_{i}$ or $y_{i}$ stands for each expression value, and $\bar{x}$ or $\bar{y}$, is the mean value of these expression values. For Spearman correlation, the vector of the expression value of each gene needs to be replaced by their ranks. So, Spearman correlation is more robust than Pearson correlation in the face of outliers and is recommended to be used in RNA-Seq data [15].

### 2.3. Module Identification

The network modules, which are groups of genes with similar expression profiles, are explored based on the topological structure of the gene co-expression network [16]. Genes in the same module tend to be functionally related and co-regulated. We implemented a module identification algorithm that was based on the node similarity measure of their relative interconnectedness coupled with the hierarchical clustering method [17].

(2) $$s_{ij}=\frac{h_{ij}+a_{ij}}{c_{i}+c_{j}-h_{ij}-a_{ij}}$$

where $s_{ij}$ is the similarity between gene i and gene j, $h_{ij}$ is the number of shared neighbour genes of gene i and gene j in the co-expression network, $a_{ij}$ is the adjacency of gene i and gene j, and $c_{i}$ and $c_{j}$ are the connectivity of gene i and gene j, respectively.

### 2.4. Function Annotation

After gene co-expression network construction and module identification, we used gene function enrichment to predict the function of novel lncRNAs or coding genes. The *p* value of the enriched function was calculated as follows.

(3) $$p=1-\overset{k-1}{\underset{i=0}{\sum }}\frac{\left(\begin{array}{c} M\\ i \end{array}\right)\left(\begin{array}{c} N-M\\ n-i \end{array}\right)}{\left(\begin{array}{c} N\\ n \end{array}\right)}$$

where *N* is the total number of background genes, *M* is the number of genes with one certain annotation in background genes, *n* is the number of neighbours of the gene to be annotated, and *k* is the number of neighbour genes with the certain annotation.

Another gene function analysis algorithm we implemented is the random walk with restart (RWR) [18], which measures each node’s relevance with respect to given seed nodes (here are genes with known function annotations) based on network propagation. The information (known gene function) is flowed in the network from seed nodes to nearby nodes until convergence. Finally, ranked information associating genes with a function of interest is attached on the nodes of the network.

(4) $$p_{k}=\alpha p_{0}+\left(1-\alpha \right)Wp_{k-1}$$

where $p_{0}$ represents our initial information on genes (seed), and *W* is a gene co-expression matrix, its columns sum was set to 1. The parameter α is the probability that the information restart to the starting node. Repeated iteration of this equation converges to a steady state. $p_{k}$ is the ranked information that associates genes with a function of interest. The implementation of RWR uses Eigen, which is a C++ template library for linear algebra (http://eigen.tuxfamily.org, accessed on 12 December 2022).

## 3. Results

### 3.1. The Main Analysis Process of GCEN

**Figure 1:**
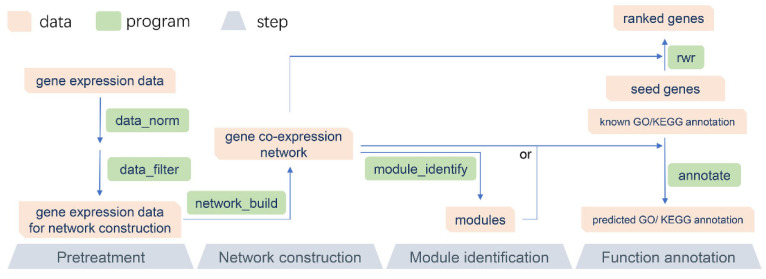
The recommended pipeline of GCEN. The recommended pipeline consists of four parts: data pretreatment, network construction, module identification, and function annotation.

The recommended pipeline of GCEN consists of four parts: data pretreatment, network construction, module identification, and function annotation (Figure 1). A README file and sample data are included in the software package, which may be of help to users. Because of its modular design, GCEN can be easily integrated into another pipeline. Moreover, the multithreaded implementation of GCEN makes it fast and efficient for RNA-Seq data.

Performing a gene co-expression network analysis requires gene expression profiles, which are usually derived from microarray or RNA-Seq. Before gene co-expression network construction, it is critical that the expression values are normalized on the same measurement scale. To remove systematic effects in the RNA-Seq data, we implemented many state-of-the-art data normalization algorithms, including quantile normalization [12], the median-of-ratios method [13], trimmed mean of M-values (TMM) [14], and housekeeping genes normalization, which have already been used in the gene differential expression analysis. Moreover, low expression of genes will bring noise to the gene co-expression network, and therefore, we needed to remove these genes according to the mean or variance of their expression values.

Next, according to the guidelines of RNA-Seq co-expression network construction and analysis, we used the Spearman or Pearson correlation coefficients directly to determine co-expression patterns between gene pairs, which perform better and are more robust [11]. Then, network modules, which are groups of genes with similar expression profiles, were explored based on the topological structure of the gene co-expression network [16]. Genes in the same module tend to be functionally related and co-regulated. We implemented a module identification algorithm based on the node similarity measure of their relative interconnectedness coupled with the hierarchical clustering method [17].

**Figure 2:**
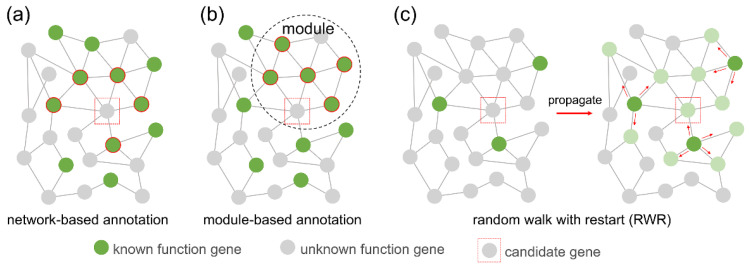
Gene function annotation methods. (**a**) Network-based function annotation. The function of an unknown gene (red box) is inferred from the function of its neighbours (green background red circle) in the network. (**b**) Module-based function annotation. The function of an unknown gene (red box) is inferred from the function of other genes (green background red circle) in the module. (**c**) Random walk with restart (RWR). Information flows in the network from the seed node (dark green) until convergence. Each node has traces of information. The rank of the node (light green) highly associated with the seed node is higher.

After gene co-expression network construction and module identification, we used gene function enrichment [19] to predict novel lncRNAs or coding gene function. According to the guilt by association (GBA) principle, we determined the function of an unknown gene by voting on its neighbour genes with known functions. These neighbour genes can be directly interacting genes in the network (Figure 2a), or they can be genes in the same module (Figure 2b). Another gene function analysis algorithm we implemented is the random walk with restart (RWR) (Figure 2c) [18], which measures each node’s relevance with respect to given seed nodes (here are genes with known function annotations) based on network propagation. The information (known gene function) is flowed in the network from seed nodes to nearby nodes until convergence. Finally, ranked information that associates genes with a function of interest is attached on the nodes of the network.

### 3.2. Performance Evaluation

Considering the recent discovery of tens of thousands of lncRNAs transcribed from the genomes of mammals and other complex organisms, we find that the number of nodes and connections in the coding-lncRNA gene co-expression network will be greatly increased in comparison with a traditional coding gene co-expression network. This poses a big challenge to the computation time and memory for constructing a co-expression network. The program GCEN is developed with C++ and has a natural advantage in terms of its performance. We tested the time and memory consumption of GCEN, FastGCN [20], and WGCNA [9] in network construction. The fastest was GCEN and it had the least memory consumption. For a network of 10,000 genes, it only takes a few seconds for network construction, and time consumption may vary because of data size and computer performance. In single thread mode, GCEN is approximately five times faster than WGCNA in calculating the correlation coefficient of gene expression. The outputs of GCEN are generated immediately after the calculation of network construction, and therefore, its memory consumption remains low without increasing significantly with the number of genes. However, WGCNA had a peak memory of more than 20GB when 40,000 genes were analysed (Table 1).

### 3.3. Data Visualization

Data visualization is essential in biological research because it presents the meaning of the data more straightforwardly. The release of GCEN does not include a plotting program as GCEN focuses on efficiently using a gene co-expression network to predict gene function. However, we did not ignore the plotting demands of biologists, and we provide a number of data visualization demos and scripts on our website. We do not offer automatic plotting instead of enlightenment to show biological significance in gene co-expression analysis.

**Figure 3:**
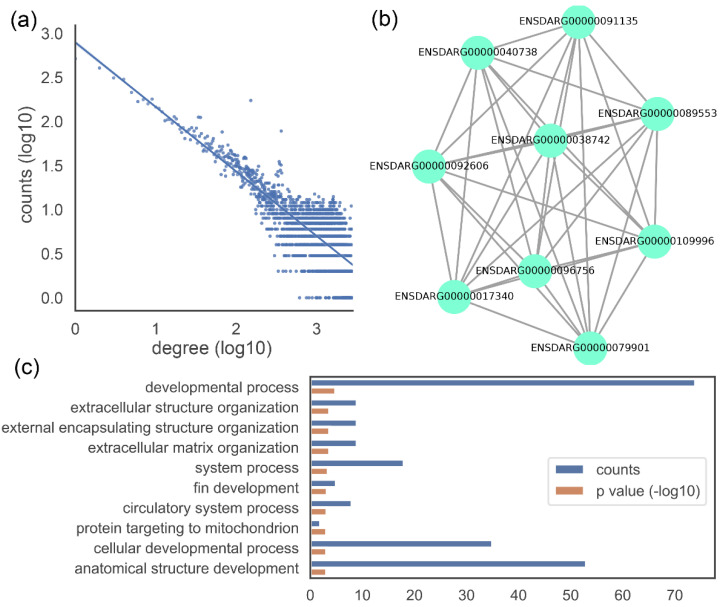
Examples of data visualization. (**a**) Network degree distribution. (**b**) Sub-network or module. (**c**) GO annotation (biological process top 10). The dataset derived from an RNA-Seq of 17 samples of zebrafish [21].

Figure 3 shows three examples of visualizations. Figure 3a shows the degree distribution of a gene co-expression network. The degree distribution of gene co-expression networks is similar to that of scale-free networks and approximately follows a power law. The majority of genes are related to a small number of other genes, while only a few genes are linked to a large number of genes. This degree distribution can be shown as a straight line on a log-log plot. Figure 3b shows the sub-network or module. We predict gene function on the basis of neighbouring genes in the network or genes in the same module. As a result, it is critical to demonstrate them graphically. Figure 3c shows GO annotations of a gene. Among the three aspects of Gene Ontology, the biological process is the most representative of gene functions and is also the most demonstrated. The code and sample data for Figure 3 are available on our website (https://www.biochen.org/gcen/visualization, accessed on 13 March 2022).

## 4. Discussion

RNA-Seq is a popular approach for studying gene expression under various biological conditions. Normalization, which scales the raw data so that different samples can be compared, is a critical step in RNA-Seq. Different normalization methods are based on different assumptions that significantly affect the downstream analysis [22]. The toolkit GCEN implements five widely used normalization methods for users to choose from. To solve the performance problem caused by the larger network with many genes in RNA-Seq, we developed GCEN in C++ for performance. A gene co-expression network analysis using GCEN can be carried out on a typical desktop or laptop computer.

The construction of a gene co-expression network is usually achieved by calculating Pearson or Spearman correlation coefficients, such as FastGCN [20], SWIM [23], and SEaCorAl [24]. WGCNA [9] first calculated the Pearson correlation coefficient, and then set a soft threshold by selecting the β parameter. Gene pairs with correlation coefficients reaching a threshold were considered to be co-expressed. Similar to WGCNA, CEMiTool [25] modified the method of selecting the β parameter, and GWENA [26] used the Spearman correlation coefficients instead of Pearson correlation coefficients. Ballouz et al. evaluated the performance of network building methods and concluded that the computationally simplest and most transparent method (direct use of the Spearman or Pearson correlation coefficients) for calculating co-expression was better than the soft thresholding method (e.g., used by WGCNA) [11]. Therefore, at present, GCEN can only build the network by calculating the Spearman or Pearson correlation coefficients, and other network building methods will be added in the future.

False positives in gene co-expression networks are a problem that cannot be ignored. Low expression of genes will bring noise to the gene co-expression network, and an empirical approach is to discard a quarter of the total number of genes. Petti et al. found that negative correlations between gene pairs may not be biologically meaningful, and further they developed the SEaCorAl to reduce spurious correlations [24]. It can be considered to keep only positive correlation in gene co-expression network. Ballouz et al. and Franziska et al. figured out that network aggregation shows better performance [11,27]. Thus, a program network_merge is included in the GCEN package that can merge two or more networks.

Our research interest focused on using computational approaches to decipher the function of long non-coding RNAs. The toolkit GCEN was also developed with this purpose in mind. Using the gene co-expression network, we obtained 7345 lncRNAs with GO annotation and 7055 lncRNAs with KEGG annotation among 13,604 zebrafish lncRNAs in our previous study [8]. More recently, we annotated 196 of 756 conserved lncRNAs in 25 flowering plants using the same method [28]. Some biologists have had concerns about the annotation accuracy. We are convinced that the prediction accuracy of the real network was higher than the random shuffled network that was used as a control [8]. There is a program calculate_accuracy in the GCEN package to calculate the accuracy of prediction. We had developed some utilities like the calculate_accuracy to assist in the gene co-expression network analysis.

Our previous study used the gene co-expression network to annotate lncRNAs on a large scale, but the gene co-expression network has a much broader application. Mathias et al. built lncRNAs co-expression networks using TCGA breast cancer data. Furthermore, they found an oncogenic lncRNA LINC00504 [29]. In another study that searched for disease-related genes, Bayraktar identified key genes of Alzheimer’s disease (AD) through AD-specific co-expression networks and genome-scale metabolic modelling of the brain in AD patients [30]. The toolkit GCEN is also suitable for such similar analysis. However, there is still some analysis that GCEN does not support, such as identification of hub genes. Recently, Paola Paci et al. developed a new algorithm called SWIM (SWItch Miner) for the discovery of a new class of hub genes from gene co-expression networks. These hub gene are called switch genes and are negatively correlated with their first nearest neighbours [24]. Then, they applied SWIM methodology to identifies novel candidate disease genes [31]. The software SWIM was originally written in MATLAB, but they have recently re-implemented it using R [32].

The toolkit GCEN still has some limitations. The command line does scare off some users. In the future, we will develop a web server based on GCEN to provide a graphical interface. At the same time, we will enhance data visualization in gene co-expression analysis. Currently, GCEN only implements two methods of network construction and one method of module identification. We will add more algorithms in the future.

Software should be well maintained during its lifecycle, and its robustness and ease of use should be improved. The software GCEN will be continuously updated on our website (https://www.biochen.org/gcen, accessed on 13 March 2022), and there is also a large amount of material on the website that can assist biologists in using it better. Moreover, connecting with our users is truly important to us. We sincerely hope that users will provide their questions and suggestions to help us improve the software.

## 5. Conclusions

We present here GCEN, an easy-to-use toolkit for gene co-expression network analysis and lncRNAs annotation. It has three notable features: it is easy to use, it has a high speed and low memory usage, and it has cross-platform application. The toolkit GCEN was primarily designed to be used in lncRNAs annotation, but is not limited to these scenarios. In the future, we will update the software and fix bugs in accordance with user feedback. We promise to maintain GCEN for 5 years or more. The software GCEN is available on our website (https://www.biochen.com/gcen, accessed on 5 February 2022) or on GitHub (https://github.com/wen-chen/gcen, accessed on 5 February 2022).

## Figures and Tables

**Table 1:** Time and memory consumption tests for network construction.

Gene Number	GCEN	FastGCN	WGCNA
10k	9.51 s/5.93 MiB	16.98 s/1.31 GiB	59.36 s/1.84 GiB
20k	37.86 s/8.50 MiB	2 m 11.59 s/5.25 GiB	3 m 47.15 s/6.36 GiB
40k	2 m 31.42 s/12.88 MiB	24 m 23.33 s/21.12 GiB	14 m 57.86 s/24.39 GiB
80k	10 m 7.70 s/21.58 MiB	Out of maximum memory	59 m 53.82 s/96.11 GiB

These tests were run on a personal computer with an Intel Core i5-10400 processor (6 cores/12 threads) and 128GB memory. The version of GCEN was 0.5.1, the version of FastGCN is v1.1, and the version of WGCNA was 1.69. The test data were a randomly generated number between 0 and 1, and each gene has 20 expression values. All tests were run in single thread mode, although GCEN has implemented multi-threading. The test data and scripts can be found on our website (https://www.biochen.com/gcen/static/benchmark.zip, accessed on 13 March 2022).

## Data Availability

GCEN is freely available under the GPLv3 license at https://www.biochen.com/gcen or https://github.com/wen-chen/gcen.
